# An alternative approach for sustainable sheep meat production: implications for food security

**DOI:** 10.1186/s40104-020-00472-z

**Published:** 2020-07-15

**Authors:** Eric N. Ponnampalam, Matthew I. Knight, Peter J. Moate, Joe L. Jacobs

**Affiliations:** 1 Animal Production Sciences, Agriculture Victoria Research, Department of Jobs, Precincts and Regions, Bundoora, VIC 3083 Australia; 2 Animal Production Sciences, Agriculture Victoria Research, Department of Jobs, Precincts and Regions, Hamilton, VIC 3300 Australia; 3 Animal Production Sciences, Agriculture Victoria Research, Department of Jobs, Precincts and Regions, Ellinbank, Victoria 3821 Australia; 4 Centre for Agricultural Innovation, School of Agriculture and Food, Faculty of Veterinary and Agricultural Sciences, The University of Melbourne, Melbourne, Victoria 3010 Australia

**Keywords:** Animal welfare, Environmental sustainability, Global demand, Livestock, Sheep meat, Summer feeding

## Abstract

**Background:**

A pelleted diet containing camelina hay (CAMH) or camelina meal (CAMM) as a supplement along with a control pellet (CONT) diet formulated with commonly available feeds during summer was used to investigate an alternative pathway for sustainable meat production. Sustainable meat production was based on a simple estimation of income from meat produced versus feed costs if animals were fed for an extended period over summer compared to early slaughter at the beginning of summer. Eighty maternal composite wether lambs (Composite) based on Coopworth genetics and 80 pure Merino wether yearlings were divided into 10 groups within breed (*n* = 8) using stratified randomisation based on liveweights. Following 1 week of adaptation to experimental diets, animals were fed experimental diets for up to10 weeks.

**Results:**

Animals were slaughtered after either 8, 9 or 10 weeks of full feeding when the average liveweight of diet/genetic combination reached a weight appropriate for either ‘heavy lamb’ or ‘heavy hogget’ production, which occurred between 8 and 10 weeks of full feeding. There was no diet × breed interactions except for dressing percentage (DP), where Composite lambs fed the CAMH diet had the greatest DP (48.1 ± 0.35) and the Merino yearlings fed the CAMM diet the lowest DP (45.8 ± 0.33). Composite lambs gained 17.6–20.3 kg and Merino yearlings gained 10.7–12.9 kg liveweight. Based on their DP, this resulted in the production of approximately 8.3–9.5 kg additional carcass weight in Composites and 4.9–5.7 kg in Merinos, which in turn produced greater profit per Composite lamb and a small profit per Merino yearling.

**Conclusions:**

Composite lambs fed CAMM and CAMH had 5% greater carcass weights at slaughter compared to the CONT group, but dietary treatments did not change carcass weight of Merino yearlings at slaughter. The extended feeding approach offered the producer an estimated economic gain of AUD $20.00 to $25.00 when yearly average prices were used (Method 1) and AUD $40.00 to $47.70 when pre- and post-summer average prices were used (Method 2) per Composite lambs, but extra carcass gain did not result in the same profit per Merino yearling. Among the Composites, the profit for animals fed the CAMH and CAMM were AUD $2.75 to $4.50 greater than CONT group when full year average prices were applied while AUD $3.50 to $5.50 greater than CONT group when pre- and post-summer average prices were applied. However, we acknowledge a combination approach of extended feeding for a portion of animals already on ground and selling the remaining animals pre-summer with joining of additional ewes is the most likely strategy. Considering the scenario of extended feeding for 3 weeks, based on the growth rates observed for Composite lambs, if an additional 2 kg carcass weight per animal can be gained for 50% of the 22 million lambs slaughtered in Australia (= 11 million animals), it would potentially supply an additional 22 million kg of lamb carcasses produced per annum. This is equivalent to producing an extra 1 million lamb carcasses per annum weighing 22 kg each. Feeding Composite lambs for an extended period and selling Merino yearlings pre-summer may be a good option due to faster growth rate of Composites that may help quick turn-over to market.

## Background

Australia is the world’s largest exporter of sheep meat and second largest producer of lamb and mutton, with the off-farm value of sheep meat up 3% from 2015–16 to 2016–17 and valued at approximately AUD $5.3 billion [1]. Lamb shipments to the Middle East, US and China for the first three quarters of 2016 accounted for 65% of Australian lamb shipments, an increase of 9% compared to 2011 and this is predicted to increase further [1]. The sustainability and viability of the sheep meat industry depends on the economic benefit to producers and processors, the social license to farm as influenced by animal welfare from conception to weaning and environmental impacts such as carbon footprint (emission of greenhouse gas) to the atmosphere as well as soil erosion. Currently, approximately 22 million lambs (under 12 months of age or an animal showing no permanent incisor teeth in wear) are slaughtered in Australia to deliver meat (lamb) for local and international markets. In addition, there are also substantial numbers of hoggets (sheep that produce meat from 12 to 18 months of age [yearlings] or animals showing at least one permanent incisor teeth) and mutton (meat from animals 18 months of age and older or from animals showing more than one permanent incisor teeth). During 2017–2018, Australia exported to international markets, 463,000 t of sheep meat of which 60% was lamb and 40% was mutton and 74% of the meat was frozen and 26% chilled product [2]. Innovative strategies are essential for the continuous, efficient use of resources that can offer sustainable animal production systems, so that Australia and other nations can benefit from the emerging changes in the livestock industry, global agriculture and trade in food and fibre.

It is foreseeable that climate variability will impact on food availability by affecting productivity of animals, crops, pastures and marine foods. As such, animal-based production research has a role to play in helping livestock producers adapt to climate variability and mitigate methane (CH_4_) emission. According to Meat and Livestock Australia [1], there has been a growing proportion of lambs processed weighing 14–16 kg carcass weight. At the same time, there were more lambs processed at approximately 26 kg carcass weight, mainly due to processors recently changing carcass specification to meet customer demands. This indicates that the current Australian national sheep flock has the potential to grow more and deliver heavier carcasses. In major sheep meat (lamb and mutton) producing regions of New South Wales, Victoria and South Australia, lambs are born during winter (June–August) and the animals are sold between late spring to early summer (October to December) at 4–5 months of age for lamb meat of trade weight (18–22 kg) carcass, or sold in late summer to early autumn (January to April) at 7–8 months of age for lamb meat of heavy weight (22 kg or more) carcass. The lambs destined for sale during October to December are raised in grazing systems while the animals intended for sale in January to April are raised in senesced pastures and flocks are typically provided with dietary supplements of pasture hay, other roughages and cereal grains. For Merino yearlings, producers also have the option to retain their animals on-farm for an extended period of feeding over summer (December to May) and slaughtering in late autumn or keeping the animals as carry-over stock to gain additional benefits from meat and wool production and slaughtering at between 12 and 18 months of age. Such decisions are dependent on feed resource availability, production purpose, market requirements and individual farmer preferences. These strategies also offer the opportunity to sell carcasses at better prices at the end of summer to meet the demand due to lower availability of animals for slaughter in that season (for example, the average price of carcass weighing 22–24 kg was AUD $5.25/kg at pre-summer whereas at end of summer during 2016–2017 the price was AUD $6.00/kg).

During the summer to winter periods of 2018 and 2019, the major sheep and cattle producing states of Australia (Victoria, New South Wales and Queensland) were under extreme drought conditions with farmers having to manage a significant shortage of traditional green feeds. As such, farmers were required to apply alternative strategies to feed stock for their businesses to remain viable. Options included using forages and/or meals produced from by-products of the vegetable oil industry or cereal production and/or pasture hay. Research conducted in Victoria has shown that lambs grazing senesced perennial pasture mainly, lucerne (*Medicago sativa* L.) during summer [3] or lambs consuming annual ryegrass (*Lolium perenne* L.) pasture-hay during autumn and fed a supplement of 10% flaxseed (*Linum usitatissimum* L) [4] can produce carcasses of 18–22 kg, classified as ‘Trade Lamb’. Another potential option is camelina (*Camelina sativa* L. Crantz), a brassica forage species, which is rich in essential fatty acids and it can withstand hot and dry conditions [5]. With a high protein and lipid concentration in its vegetative parts [6], camelina has the potential to improve animal performance and carcass yield without adversely affecting meat quality. Camelina seed or meal has been recently introduced as a feed supplement to improve growth rate and carcass value in pigs and poultry [7], but there is no information available regarding its effects on sheep growth and carcass production. We compared the effects on estimates of carcass weight, meat production and profit of the current farm scenario of slaughtering lambs in pre-summer versus an alternative scenario of feeding lambs for an extended period over summer. For the extended feeding scenario, we investigated whether the inclusion of a pelleted diet containing camelina hay (CAMH) or a pelleted diet containing camelina meal (CAMM) as a supplement to a control pelleted diet (CONT) would increase liveweight gain, dressing percentage (DP) and estimated carcass value of sheep. We hypothesise that due to increased liveweight gain and carcass weight, the estimated economic benefit would be greater when CAMH or CAMM were fed for an extended period over summer compared to early slaughter at the beginning of summer. Implications of these findings for the Australian sheep meat industry are discussed in terms of meat supply to national and international markets. Maternal Composite (Composites) lambs and Merino yearlings (Merinos) were used to evaluate lamb and mutton production, respectively.

## Methods

### Experimental design and dietary treatments

**Fig. 1:**
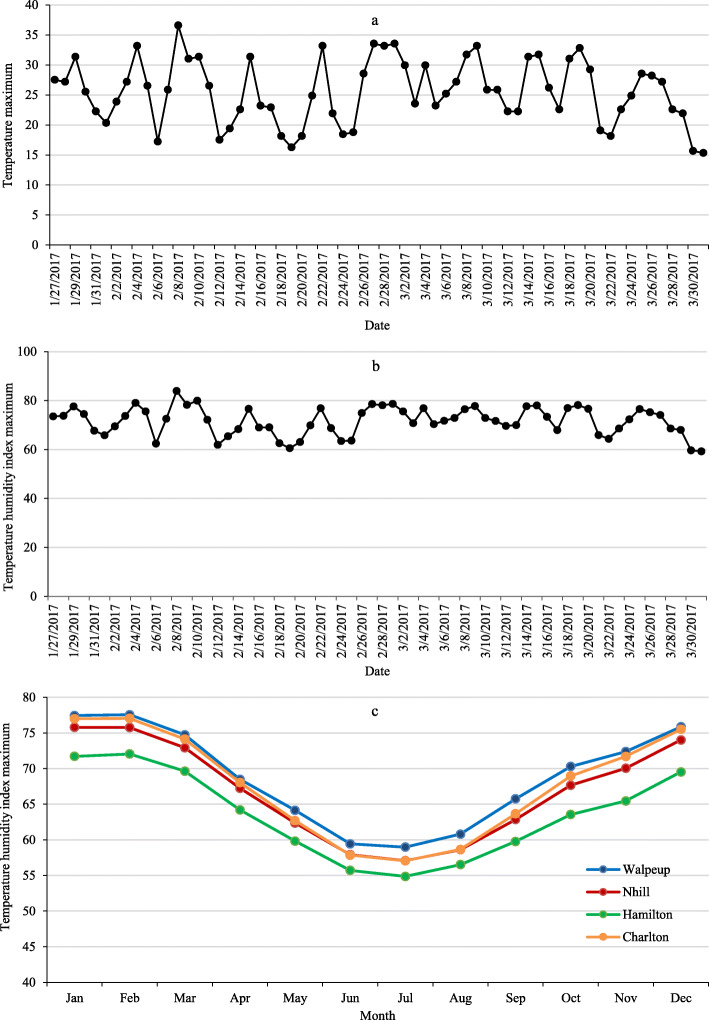
Maximum daily temperature (**a**), maximum daily temperature humidity index (**b**) of animal house facility in Hamilton, where the animal feeding experiment was undertaken and maximum monthly temperature humidity index (**c**) recorded during the period of 2013 to 2017 for four sheep producing regions (Walpeup, Nhill, Hamilton and Charlton) in Victoria

Maternal Composite (Composite) wether lambs (a composite mixed sheep breed comprising of Coopworth, Border Leicester, East Friesian, Booroola genetics bred for maternal and meat production characteristics) and pure Merino wether yearlings were used to examine experimental diets for ‘Heavy lamb’ and ‘Heavy hogget’ production during Summer 2017 (January to March) in south-west Victoria. In this experiment, faster growing Composite lambs and slower growing Merino yearlings were used to produce lamb (meat) and mutton, respectively. This is because faster growing Composites (crossbred) are mainly reared for meat production. While, Merino sheep are either bred for replacement ewes in the breeding flock or as carry-over stock to gain additional benefits from meat and wool production. These animals are generally slaughtered at between 12 and 18 months of age. This farming practice is common by Merino sheep producers because Merinos grow slower and achieve market weight at older age than crossbred sheep [8, 9]. The study was approved by the Agricultural Research and Extension Animal Ethics Committee, DEDJTR (AEC Code No: 2016–17) and all procedures were conducted in accordance with the Australian Code of Practice for the Care and Use of Animals for Scientific Purposes [10]. The rainfall and temperature conditions were consistent with the long-term data recorded in sheep producing regions of Victoria. Figure 1 shows the maximum daily temperature (Fig. 1a) and maximum temperature humidity index (Fig. 1b) recorded during the experimental period at the Animal house facility, and the maximum daily temperature humidity index (THI, Fig. 1c) recorded during the period of 2013 to 2017 for four sheep producing regions of Walpeup, Nhill, Hamilton and Charlton in Victoria.

Three experimental diets: control pellets (CONT), a diet containing camelina hay pellets (CAMH) and a diet containing camelina meal pellets (CAMM) were selected to examine the impact of diets on lamb (Composite lamb) and hogget (Merino yearling) production. Diets were formulated using ingredients available in the major sheep producing regions of Victoria and South Australia during the summer period. During summer, sheep producers in south-west Victoria generally rely on the grazing of dried senesced pastures or cereal hay and stubble as the basal diets with available cereal or legume grains provided as supplements. The ingredients of the CONT diet were chosen so that the nutrient composition was similar to diets that producers feed to their sheep over this period. The camelina hay used for CAMH diet was grown in south-west Victoria as a forage crop, harvested at mid-bloom stage and dried under sun. The camelina meal used for CAMM diet was the residue after oil had been extracted from camelina seeds. The dietary ingredients used for the three treatments are shown in Table 1. All dietary ingredients (grain and hay) were smashed using a hammer mill and a 5-mm screen used to control particle size. Included in all pellets of all treatments was a binding agent containing 10 kg lime and 10 kg bentonite per tonne (1,000 kg) of pellets. The pellets were made by heat pressing followed by cooling and were produced by a commercial feed manufacturer (J.T. Johnson & Sons & TMR Feed Solutions, South Australia). Pellets of all dietary treatments were of 9 mm in diameter and 15 mm in length.

**Table 1:** Dietary ingredients and nutritive characteristics of pelleted diets used for the full feeding of maternal composite lambs and Merino yearlings (hoggets)

Items	Dietary ingredients used, %		
	Control pellet (CONT)	Camelina hay (CAMH)	Camelina meal (CAMM)
Camelina meal	0	0	8
Lupins	30	30	22
Barley grain	20	10	20
Oat grain	5	15	5
Oaten hay	45	0	45
Camelina-Oat-Barley hay, 33:33:33 w/w/w	0	45	0
Nutritive characteristics of diet^1^			
Dry matter, %	89.35	88.85	88.90
Crude protein, % DM	14.80	15.20	14.93
Metabolisable energy, MJ/ kg DM	10.80	10.83	11.23
Crude fat, % DM	2.11	2.91	3.65
Acid detergent fibre, % DM	19.03	19.93	17.78
Neutral detergent fibre, % DM	34.23	34.03	31.18
Lignin, % DM	4.30	4.20	4.13
Phosphorous, % DM	0.47	0.42	0.52
Potassium, % DM	1.47	1.42	1.52
Sulphur, % DM	0.25	0.21	0.26

^1^*DM* Dry matter, *ME* Metabolizable energy, *ADF* Acid detergent fibre, *NDF* Neutral detergent fibre

Eighty Composite weather lambs (average 33.7 ± 3.2 kg) and 80 Merino weather yearlings (average 40.1 ± 1.9 kg) were selected from a research flock with known pedigree from Agriculture Victoria Research, Hamilton Centre, Victoria. At the commencement of the experiment, the ages of Composite lambs and Merino yearlings were 4 and 15 months ±15 days, respectively. Both breeds consisted of 75% twin born lambs and 25% single born lambs. Composite lambs and Merino yearlings were equally distributed to pens based on birth type (single or twin). Animals were managed in 20 pens in the Animal house facility, each of which could hold up to 14 lambs of 45–50 kg liveweight. Each pen contained two feed troughs of 21 cm width × 84 cm length × 51 cm height, with each capable of holding approximately 10 kg DM adequate for 4 sheep. Animals always had free access to water. Animals were divided into 10 groups within each sheep type by stratified randomisation based on liveweight and allocated to a 2 × 3 factorial fully randomised design with pens as the experimental unit (*n* = 8 animals per group/pen). For each breed, there were 3 pens fed the CAMH diet, 3 pens fed the CAMM diet and 4 pens fed the CONT diet.

### Feeding and measurements

Two weeks prior to introduction of animals to the Animal house and while the animals were still in the paddock, both Composite lambs and Merino yearlings were trained to consume a standard commercial pellet containing 9.6 MJ/kg DM and 9.8% CP (Heywood Stockfeeds, Victoria). During the first week of adaptation to pens, animals were offered the same standard commercial pellet at approximately 2.0 kg DM/d per head. In the second week, animals were given the same commercial pellet in decreasing rates; while experimental diets were increased gradually from 200 g/d per head to an *ad libitum* amount. Animals were then fed the experimental diets *ad libitum* until time for slaughter. Feed troughs were designed to minimise feed wastage, and hence feed intake per Composite lamb or Merino yearling were available as group-based average for each pen as a unit.

Feeds offered were recorded daily and refusals measured weekly. Samples of the three diets were collected weekly over the course of the experiment and the samples bulked by treatment. These samples were dried at 100 °C, ground using a UDY Cyclone Mill (model # 3010–019) equipped with 1 mm sample screen and then analysed for chemical composition determined by near infrared spectrometry (NIR, ACE Laboratories Pty. Ltd., Bendigo, Victoria, Australia). NIR prediction equations developed by Cumberland Valley Analytical Services (www.foragelab.com) using in house chemistry and NIR spectra using WinISI (Foss) chemometric software were used to determine the chemical constituents as reported in detail [11]. The chemical compositions of the three experimental diets are shown in Table 1. Diets were designed to achieve at least 150 g/d of LWG for both Merino sheep and Composite lambs.

A weekly feed sample was also collected from each pen, combined and a homogeneous feed sample was used for the determination of DM content by drying them at 100 °C for 24 h. These DM values were used to calculate the weekly average dry matter intake (DMI) for each dietary treatment. Liveweights of animals were recorded at the commencement (week 0), then weekly throughout the experimental period and 1 d prior to slaughter using a weigh crate (Pratley 3-Way Manual Drafter, Pratley Industries Ltd., Temuka, New Zealand) and scales (Tru-Test MP600, Tru-Test Livestock Management, Victoria, Australia). These data were used to obtain growth rate (LWG/d), total liveweight gain and liveweight at slaughter for the entire feeding period.

### Slaughter procedure and measurements of carcass traits

Animals were slaughtered when the average liveweight of diet/genetic combination reached a weight appropriate for ‘heavy lamb’ and ‘heavy hogget’ production (i.e., 50–52 kg or above to achieve carcass weight of 22 kg or above), which occurred between 8 and 10 weeks of full feeding, depending on treatment group. For 8 and 9 weeks slaughter, heavier weight animals were selected in equal numbers from each pen in order to maintain an even distribution of animals across treatments. Animals were transported for approximately 3 h by highway surface travel to a commercial abattoir and after 18 h in lairage, were slaughtered with carcass weight and GR fat depth (total muscle and fat tissue depth at 11^th^/12^th^ rib intersection, 110 mm from the midline) recorded at 1 h post-slaughter.

### Calculation of total feed cost and profit

The estimated economic benefits of feeding camelina based diets to Composite lambs and Merino yearlings were calculated in two ways based on an estimation of income from carcass sale price over feed costs. For both ways, total feed intake for the entire feeding period was calculated using the weekly feed intake recorded for each pen over the 8–10 weeks feeding, as the animals being slaughtered in a series after 8, 9 or 10 weeks of feeding appropriate for ‘heavy lamb’ and ‘heavy hogget’ production. The total feed cost for the entire study was obtained by multiplying the total feed intake (kg) per pen by the cost of ration per kg based on the prices of ingredients used for the formulation of each treatment diet. The weekly feed intake, total feed intake and feed cost calculated for 20 pens for the entire study were then used for the statistical analysis in order to obtain the dietary treatment means for both animal types.

### Method 1: estimated economic benefit calculated using 5-year Victorian state average price (AUD $/kg carcass) obtained for each carcass range classification

For each animal, carcass weight was obtained at the time of slaughter. These individual carcass weights were then used to obtain pen averages for all 20 pens. These pen averages for carcass weights were then multiplied by the 5-year Victorian state average price per kg carcass (AUD $/kg carcass) to determine the real total carcass value per pen (AUD $/sheep/pen). The estimated initial carcass weight for each pen was determined using the average initial liveweight for each pen recorded at the commencement of the experiment, multiplied by their final dressing percentage (DP = hot carcass weight/ liveweight at slaughter) obtained at slaughter. The estimated initial carcass weight for all 20 pens was then multiplied by the 5-year Victorian state average price per kg carcass (AUD $/kg carcass) to obtain the estimated total carcass value (AUD $/sheep/pen) at the start of the experiment. The carcass classifications and carcass weight ranges applied were light lamb (12–18 kg), trade lamb (18–22 kg), heavy lamb (22 kg+), Merino (14–18 kg), Mutton from hogget (18–24 kg) and Mutton from old sheep (24 kg+), respectively. The estimated profit for each pen was determined by subtracting the average total feed cost for each pen for the entire study from the difference between real carcass value at slaughter and estimated initial carcass value, calculated for each pen.

### Method 2: estimated economic benefit calculated using 3-year average carcass price (AUD $/kg carcass) obtained during pre-summer and post-summer for each carcass range classification

At the time of slaughter, the prices obtained for Composite lambs and Merino yearlings were AUD $6.00 and $4.80 per kg of carcass, respectively. However, for the calculation of initial carcass value (pre-summer) and final carcass value (post-summer) for each pen (AUD $/sheep/pen), we have used the averages of 3-year (2016–2018) carcass prices available for pre-summer and post-summer in Victoria state, where the study was conducted. The carcass classifications and weight ranges applied were the same reported as above for method 1 calculation. Accordingly, the estimated initial carcass value for each pen (AUD $/sheep/pen) was calculated as estimated initial carcass weight per pen multiplied by 3-year average carcass price obtained for pre-summer. Similarly, the final carcass value for each pen ($/sheep/pen) was calculated as the final carcass weight per pen obtained at the time of slaughter multiplied by 3-year average carcass price obtained for post-summer. The estimated profit for each pen was determined by subtracting the average total feed cost for each pen for the entire study from the difference between final carcass value per pen and estimated initial carcass value per pen. We note that the method 1 approach is fairer than the method 2 approach because it is not influenced by the vagaries of market prices (AUD $/kg of carcass) and that end of summer market prices may not always be greater than market prices in pre-summer.

### Calculation of methane emissions using different production scenarios

The modelling described in this section has been used to put the current experiment results and conclusions in context, in terms of the effects that different feeding strategies could have on CH_4_ emissions. Methane emissions were calculated using CH_4_ yields (g CH_4_/ kg DMI) obtained for sheep reported by Swainson and others [12] and applying different production scenarios. Specifically, for sheep greater or less than 1 year of age, 20.9 g and 16.8 g CH_4_ emissions per kg DMI were used for all scenarios in the modelling, respectively. The CH_4_ yields were obtained under grazing conditions at pasture [12]. The theoretical output of CH_4_ emission scenario modelling was to obtain an additional 22 million kg of lamb carcasses per annum by feeding the 11 million lambs already on ground (50% of the current 22 million lambs slaughtered) for an extra 3-week to attain an additional 2 kg carcass weight per animal as opposed to producing 1 million extra new lambs in the following year delivering 22 kg carcass over five months of feeding as described in four scenarios below. To achieve these outcomes, scenario 1 determined the amount of CH_4_ produced from generating 1 million extra lambs per annum at a target carcass weight of 22 kg. We assumed to generate this progeny, an additional 800,000 ewes are required, and these animals would consume 3 kg DMI per day for 120 d of fattening before mating, 3 kg DM per day for 150 d of pregnancy and 3 kg DM per day for 60 d of lactation to weaning. The 1 million extra lambs would themselves consume 2 kg DM of feed per day over 5 mo to achieve the target carcass weight of 22 kg. This scenario assumes a conception rate of 150% and includes an allowance for the 15–20% lamb losses that occur during conception to birth and to weaning. Variations on scenario 1 included varying the assumed DMI/d from 3.0 to 2.5 kg DMI/d for ewes, while maintaining 2 kg dry matter intake per day for lambs (Scenario 2). Scenario 3 maintained ewe DMI/d at 3.0 kg and varied lamb DMI from 1 kg DMI/d for the first 2.5 months and 2 kg DMI/d for the last 2.5 months before slaughter. Scenario 4 reduced ewe DMI/d from 3.0 kg to 2.5 kg and maintained lamb DMI/d similar to Scenario 3 (Fig. 3). These 4 different scenarios were compared to supplementary feeding of 11 million lambs already on ground (50% of Australia’s 22 million annual lamb slaughter population) for an additional 3-week at 2 kg DMI/d to gain an additional 2 kg carcass weight per animal. The model reports the difference in CH_4_ emissions produced (i.e., reduction in CH_4_ emission) between each scenario modelled for producing another 1 million new lambs by joining an additional 800,000 ewes every year versus extended feeding of 11 million lambs already on ground for 3 weeks to produce an additional 22 million kg of lamb carcasses per annum.

### Statistical analyses

As animals were slaughtered after either 8, 9 or 10 weeks of full feeding as they reached the target liveweight, the feed intake was determined for each pen up to the slaughter. These data were then used to calculate the total feed consumption per pen (as explained in detail above under calculation of feed cost and profit). Estimated carcass value per pen and real carcass value per pen were obtained using 5-year average data and 3-year average for pre-summer and post-summer data in Victoria state with prices applied for each carcass weight range classification (as explained in detail above under calculation of estimated economic benefit in methods 1 and 2). For liveweights and carcass traits analysis, data collected from individual animals were used for statistical comparison (animal as experimental unit). For economic benefit statistical comparison, the parameters of weekly feed intake, total feed intake, total feed cost, total carcass value, carcass price difference (real carcass value - estimated initial carcass value) and profit (carcass price difference - total feed cost) were analysed using the pen average data calculated for all 20 pens (pen as experimental unit). Data were subjected to ANOVA procedures in GenStat 18 [13] for statistical analyses. Treatment means were determined for each dietary treatment (CONT, CAMH, CAMM) within each animal type (Composite and Merino) and the diet × animal type interaction. The differences were declared as significant at *P* < 0.05 but all *P* values are presented in Tables.

## Results

### Feed intake, growth performance and carcass characteristics

**Fig. 2:**
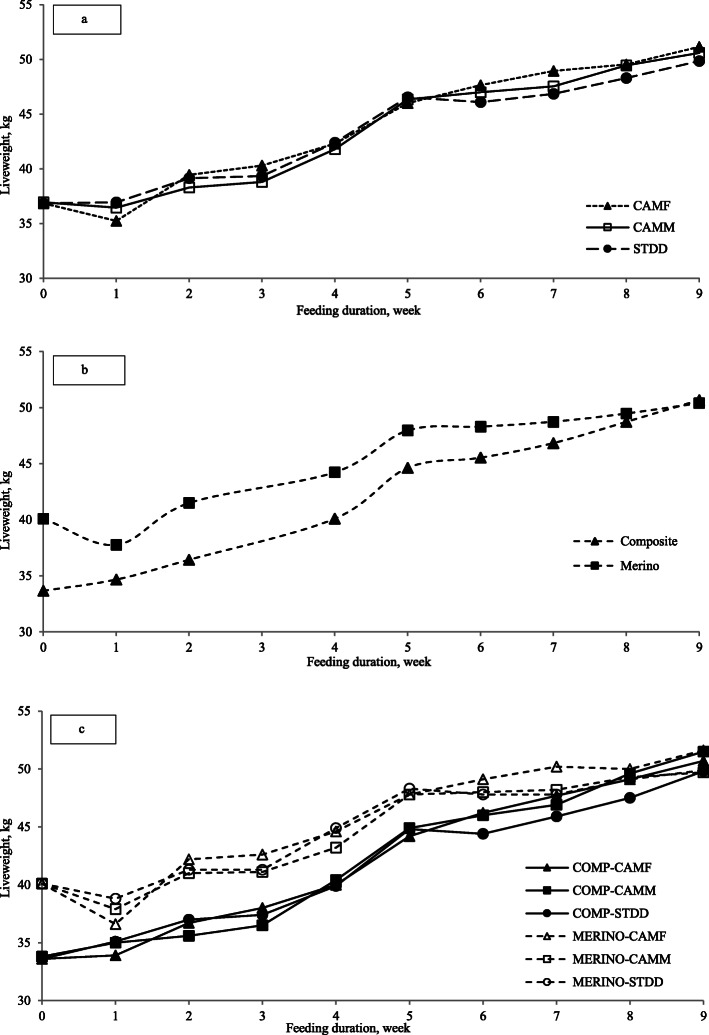
Weekly average liveweight of lambs for diets (**a**), breeds (**b**) and diet × breed interaction (**c**) over the experimental feeding period. Denotations: CAMH = camelina hay pellet diet; CAMM = camelina meal pellet diet; CONT = STDD = control pellet diet; COMP = Composites

There was no diet × breed interactions except for DP (*P* < 0.008) where Composite lambs fed the CAMH diet had the greatest DP and Merino yearlings fed the CAMM diet the smallest DP (Table 2). For main effects, diet had no effect on DP but there was a breed effect with Composite lambs differing from Merino yearlings (*P* < 0.001, 48.2% vs. 46.8%). There was a breed effect observed for DMI with smaller (*P* < 0.001) DMI for Merino yearlings than the Composites but there was no effect of diet on DMI (*P* > 0.05). Composite lambs fed camelina diets grew 20–40 g/d faster (*P* < 0.04) than those offered the CONT diet, but this was only observed in Merino yearlings when fed the CAMH diet (Fig. 2a-c). Composite lambs fed CAMM and CAMH had 5% greater (*P* < 0.03) carcass weights at slaughter compared to the CONT but dietary treatments did not alter carcass weight of Merino yearlings (Table 2). For diet and breed main effects, the carcass weight for CAMM and CAMH were greater (*P* < 0.03) than CONT diet with values of 24.3, 24.5 and 23.6 kg, respectively, while Merino yearlings produced smaller carcasses than Composites (23.5 vs. 24.7 kg; *P* < 0.001). The dietary treatment or animal type did not affect subcutaneous fat as assessed by GR and all carcasses were within the range suitable for Australian national and export markets (fat score 2–4 = 10–20 mm thickness).

**Table 2:** Initial liveweight, liveweight at slaughter, hot carcass weight, dressing percentage and GR fatness (GR = total muscle and fat tissue depth at 11^th^/12^th^ rib intersection, 110 mm from the midline) of maternal composite (Composite) lambs and Merino yearlings fed camelina hay (CAMH), camelina meal (CAMM) supplemented pellet diet or control pellet diet (CONT)^1^

Items	Composite lambs			Merino yearlings (hogget)			SED	*P*-value		
	CONT	CAMH	CAMM	CONT	CAMH	CAMM	Diet × Breed	Diet	Breed	D × B
Initial liveweight, kg	33.6	33.6	33.8	40.1	40.1	40.1	0.16	0.78	0.001	0.51
Liveweight at slaughter, kg	51.2	52.8	54.1	50.8	52.9	51.0	0.99	0.05	0.06	0.09
Liveweight gain, kg	17.6	19.1	20.3	10.7	12.8	10.9	0.91	0.02	0.001	0.10
Liveweight gain per day, g	254	276	292	154	186	158	14	0.04	0.001	0.13
Carcass weight, kg	23.9	25.3	25.2	23.3	23.7	23.3	0.43	0.03	0.001	0.12
Dressing percentage, %	46.8^b^	48.0^c^	46.6^b^	46.1^ab^	45.0^a^	45.8^a^	0.49	0.63	0.001	0.008
GR fatness, mm	16.6	17.8	17.1	15.7	17.5	15.44	0.98	0.10	0.13	0.69

^1^Values within a row followed by different letters are significantly different at *P* = 0.05. *SED* Standard error of difference, *D × B* Interaction of diet by breed

### Carcass classification

Carcass classification description was given as observational data based on the numbers counted in each weight range, and no direct statistical analyses were performed on these data (Supplementary Table 1). However, these carcass weight data were used for the statistical analyses of estimated economic benefits (carcass value initial and final, and profit) based on the carcass prices available for each weight classification as explained under method 1 and 2 above. Most of the carcasses from Merino yearlings irrespective of diet were in the heavy weight range, 22–25 kg. Composite lambs had most carcasses classified in the extreme heavy weight range, > 25 kg for CAMH and CAMM treatments, with CONT group producing heavy weight range carcasses that were similar to the Merino yearlings from all three treatment groups (Supplementary Table 1).

### Estimated economic benefit

Based on how economic benefit (profit) was calculated, the figures were different for Composite lambs and Merino yearlings fed the same diet over the feeding period. The profit for Composite lambs fed all three diets were greater than the profits estimated for Merino yearlings fed the same diets (Table 3). The profit was approximately 50% greater for Composites and even more (~ 100% greater) for Merinos, when pre-summer and post-summer average carcass prices (AUD $/kg carcass) were applied for calculation (method 2) than using all year average carcass prices (method 1). If these Composite lambs and Merino yearlings were just sold at the pre-summer season without an extended feeding approach, the estimated carcass weights would have been ~ 16 kg for Composite lambs and ~ 18 kg for Merino yearlings based on their final DP. With the extended feeding over summer, Composite lambs produced approximately an extra 9 kg of estimated carcass weight and Merino yearlings an extra 5 kg of estimated carcass weight, based on pen average calculation. Among the Composites, the profit for animals fed the CAMH and CAMM were AUD $2.75 to $4.50 greater when full year average prices were applied (method 1) while AUD $3.50 to $5.50 when pre- and post-summer average prices were applied (method 2) than those fed the CONT diet.

**Table 3:** The economic benefit (profit margin) calculated based on feed consumption, liveweight gain and carcass weight (estimated and real) of maternal composite (Composite) lambs and Merino yearlings (hogget) fed camelina hay (CAMH), camelina meal (CAMM) supplemented pellet diet or control pellet diet (CONT) for all animals up to slaughter^1^

Items	Composite lambs			Merino yearlings (hogget)			SED	*P*-value		
	CONT	CAMH	CAMM	CONT	CAMH	CAMM	Diet × Breed	Diet	Breed	D × B
Daily feed intake, kg	2.18	2.15	2.19	2.02	2.06	2.04	0.05	0.85	0.001	0.51
Total feed intake, kg	137.5	135.3	138.4	127.0	129.8	128.7	3.39	0.85	0.001	0.51
Feed price, AUD $/ kg	0.18	0.19	0.21	0.18	0.19	0.21	NA	NA	NA	NA
Total feed price, AUD $	24.75	25.65	29.00	22.85	24.65	27.00	0.66	0.001	0.001	0.51
Method 1^2^										
Estimated carcass weight initial, kg	15.8	16.1	15.8	18.4	18.0	18.4	0.23	0.79	0.001	0.07
Estimated carcass value initial, AUD $	88.50	90.40	88.35	68.15	62.50	67.55	2.01	0.43	0.001	0.04
Real carcass weight at slaughter, kg	23.9	25.3	25.2	23.3	23.7	23.3	0.43	0.03	0.001	0.12
Real carcass value at slaughter, AUD $	134.2^b^	141.6^c^	141.1^c^	86.0^a^	82.6^a^	85.8^a^	2.75	0.25	0.001	0.04
Carcass value difference (real minus estimated), AUD $	45.65	51.10	52.70	17.80	20.05	18.30	2.00	0.02	0.001	0.11
Profit 1 (Carcass value difference - total feed price), approximate AUD $	20.90	25.50	23.65	−5.00	−4.60	−8.70	2.05	0.15	0.001	0.11
Method 2^3^										
Estimated carcass weight pre-summer, kg	15.8	16.1	15.8	18.4	18.0	18.4	0.23	0.79	0.001	0.07
Estimated carcass value pre-summer, AUD $	85.50	87.40	85.40	61.90	59.50	60.75	1.29	0.79	0.001	0.10
Real carcass weight post-summer, kg	23.9	25.3	25.2	23.3	23.7	23.3	0.43	0.03	0.001	0.12
Real carcass value post-summer, AUD $	151.90^b^	160.30^c^	159.70^c^	90.20^a^	89.50^a^	90.00^a^	2.36	0.05	0.001	0.03
Carcass value difference (pre-summer minus post-summer), AUD $	66.40	72.90	74.35	28.30	29.95	29.30	2.38	0.03	0.001	0.13
Profit 2 (Carcass value difference - total feed price), approximate AUD $	41.65	47.25	45.30	5.50	5.30	2.25	2.44	0.28	0.001	0.13

^1^Values were the average of all 80 Composite lambs and 80 Merino yearlings used in this study, calculated on pen (*n* = 20) basis with each pen had 8 Composites (*n* = 8) and 8 Merinos (*n* = 8), respectively. Values within a row followed by different letters are significantly different at *P* = 0.05.

*SED* Standard error of difference. *D × B* Interaction of diet by breed, *NA* Not applicable

^2^Method 1: Profit calculated using 5-year Victorian state average price per kg carcass (AUD $/ kg carcass) for each carcass classifications and carcass weight ranges

^3^Method 2: Profit margin calculated using 3-year average price per kg carcass received pre-summer and post-summer (AUD $/ kg carcass) for each carcass classifications and carcass weight ranges

### Modelled methane emissions based on different sheep meat (lamb) production scenarios

**Fig. 3:**
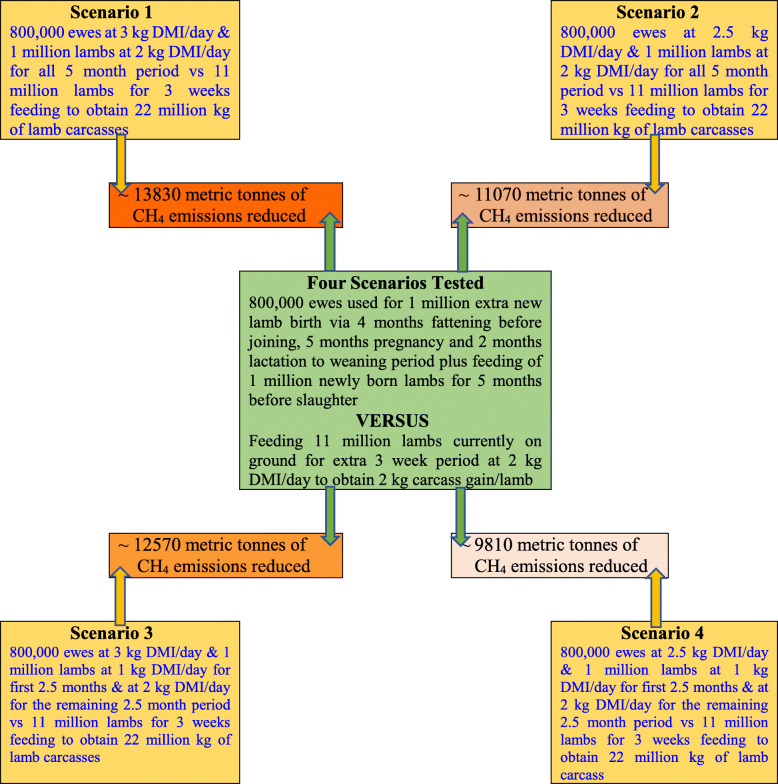
A schematic diagram showing the calculation of methane (CH_4_) emissions considering 4 scenarios that compares different amounts of dry matter intakes (DMI) for 800,000 ewes and their 1 million lamb progeny at 150% conception rate (i.e., 400,000 carry twins and 400,000 carry singles with 20% loss from gestation to weaning). For all 4 scenarios, the values for ewes were calculated at 3.0 kg or 2.5 kg DMI per day and for their progeny lambs at 1 kg or 2 kg DMI per day, but DM intakes of 11 million lambs were calculated at 2 kg DMI per day for all the 3-week of additional (extended) feeding. Methane emissions calculation was done based on the rate obtained for sheep grazing fresh grass dominated pasture reported by Swainson et al. [12] using 20.9 g CH_4_/kg DM intake for sheep more than 1 year old and 16.8 g CH_4_/kg DM intake for sheep less than 1 year old.

Based on different production scenarios investigated to produce an additional 22 million kg of lamb carcasses per annum (Fig. 3), the reduction in CH_4_ emissions vary with each feed intake category. For example, an estimated additional 13830 metric tonnes of CH_4_ will be emitted to the environment if another 1 million new lambs weighing 22 kg carcass are produced by joining an additional 800,000 ewes every year and assuming ewes consume 3 kg DMI per day for the 120 d of fattening before mating, 150 d of pregnancy and the 60 d of lactation to weaning and, assuming the 1 million extra lambs would themselves consume 2 kg DM per day over 5 months (scenario 1). Assuming if ewes eat 2.5 kg DM/d instead of 3.0 kg DM/d and assuming lambs eat 2.0 kg DM/d also resulted in a theoretical reduction of 11070 metric tonnes in CH_4_ emissions (scenario 2). If however, we assume that these additional newly born 1 million lambs would consume 1 kg DMI/d in the first 2.5-month and then 2 kg DMI/d for the remaining 2.5-month period and, assuming ewes eat 3.0 kg DM/d through fattening to lamb weaning stage, an estimated additional 12570 metric tonnes of CH_4_ will be emitted to the environment (scenario 3). Assuming if ewes eat 2.5 kg DM/d instead of 3.0 kg DMI/d and, assuming lambs eat 1 kg DM/d in the first 2.5-month and then 2 kg DMI/d for the remaining 2.5-month period also resulted in a theoretical reduction of 9810 metric tonnes in CH_4_ emissions (scenario 4). As mentioned earlier, these estimated CH_4_ emissions reduction were identified when compared to feeding the 11 million lambs already on ground (50% of the current 22 million lambs slaughtered) for an extra 3-week to attain an additional 2 kg carcass weight per animal. However, feeding 11 million lambs at 2 kg DMI/d for an additional 3-week produces less CH_4_ emissions than growing an additional 1 million lambs for 5 months.

## Discussion

Although the Merino yearlings on average were heavier at the start of the experiment, their smaller DMI compared with that of the Composite lambs resulted in smaller LWG and carcass weights for the Merino yearlings. The average daily liveweight gain by the Composite lambs (274 ± 25 g/d) was greater (*P* = 0.001) than that of the Merino yearlings (166 ± 19 g/d). There are two likely reasons for the slower growth rate with Merino yearlings: 1) Merino yearlings were 15 months old so the growth rate might be slow due to their maturity as opposed to the faster growth rates of the younger Composite lambs [9, 14, 15]; 2) Merinos have different basal and hormone stimulated energy metabolism compared with the faster growing first cross meat producing sheep [16] and the DMI and partitioning of dietary energy into maintenance and body gain might be different.

On average, the 9 week of feeding experimental diets allowed Composite lambs and Merino yearlings to grow a further 17.6–20.3 kg and 10.7–12.9 kg, respectively. This resulted in the production of an additional 8.3–9.5 kg estimated carcass in Composites and a 4.9–5.7 kg estimated carcass in Merino yearlings based on their 46% to 48% DP. This offered the producer an estimated economic gain of AUD $20.00 to $25.00 when yearly average prices are used (method 1) and AUD $40.00 to $47.70 when pre- and post-summer average prices are used (method 2) per Composite lamb. In contrast, producers of Merino yearlings could expect AUD $4.60 to $8.70 loss when yearly average prices are used and AUD $2.25 to $5.50 when pre- and post-summer average prices are used per Merino yearling. Our results suggest that the extended feeding approach will allow producers to gain extra AUD $2.75 to $5.50 profit per composite lamb for each kg of carcass gain based on the yearly average prices or pre- and post-summer average prices used. In the meantime, the Australian red meat industry has revised the definition of lamb to young sheep under 12 months of age or sheep which do not have any permanent incisor teeth in wear. This change in definition will enable many Merino yearlings to be classified as young sheep and likely result in better economic gain than we have observed for Merino sheep here because prices are slightly higher for meat from young Merino sheep (yearling) than older Merino sheep (mutton).

Traditionally the carcass price for faster growing crossbred meat animals is greater than that for slower growing Merino sheep at a particular weight range as consumers in some countries such as Australia, New Zealand and Europe prefer younger animals due to the better carcass composition (more muscle: less fat ratio). However, specific market segments from Asia and middle Eastern countries prefer meat from older animals such as hogget (18 to 24 months old) or mutton (more than 24 months old) due to the flavor and taste associated with fat from meat, since older animals deposit more fat in the carcass. Here we note that the food preparation methods also differ between different ethnic groups and different countries. When carcass composition was determined for a subset of animals (30 Composites and 30 Merinos) using a dual energy X-ray absorptiometry [17], carcasses from Composite lambs had 3% lower (*P* < 0.001) carcass fat and 0.7% ash (bone mineral concentration) and 2.3% greater (*P* < 0.001) carcass lean than the carcasses from older Merino yearlings, respectively. Both camelina diets produced carcasses that were around 5% heavier than those of the control group in each animal type. This extra carcass weight was due to extra fat accretion but not the lean accretion while the GR fat score was below 20 mm (fat score 4), a score acceptable to national and international markets. In addition, colour stability of fresh meat was unaffected by diet, with 72 h retail colour considered acceptable for consumers [17].

Australia is known to produce clean and green agricultural food commodities [18]. It is worth noting that the ME and CP concentrations of all diets used in the present experiment were 10–11 MJ/kg DM and 14–15% CP and categorized as diets containing moderate nutritive characteristics. The ingredients used to formulate the diets in the present study were suitable to achieve heavy weight (22–24 kg) to extreme heavy weight (> 24 kg) lamb and yearling carcasses during summer or autumn. On average, the carcass weights from all dietary groups in the current experiment were heavier than those observed in recent studies conducted in Victoria over Summer to Autumn under grazing or intensive feeding with the carcass weighing 18–22 kg classified as ‘Trade lamb’ [3, 4, 19]. The findings of the current experiment indicate that there are potential options for extended forage-based strategies, even strategies with shorter feeding periods and lower feed costs to produce ‘heavy weight’ or ‘extreme heavy weight’ carcasses with acceptable meat quality, when green forage availability is limited.

Over the last two decades, the world has seen significant changes in global agriculture and food trade, particularly in the Asia Pacific and sub-Saharan (Africa) regions in terms of population economic shift and consumption of increased amounts of animal and horticulturally based foods imported from developed countries [20, 21]. These phenomena introduce complex challenges for food security in the animal-plant production sector. Currently, Australia slaughters around 22 million lambs per annum for trade meat sent to national and international markets. Meat and Livestock Australia predicts this will continue to grow to 23 million lambs in the coming years [1]. In addition, Australia exports large amounts of sheep meat from hoggets and mutton to Asian and middle Eastern countries generating significant income. This poses at least one hypothetical dilemma for the Australian sheep industry: Is it better to maintain the current Australian slaughter at 22 million lambs and yearlings per annum with an additional period of on-farm feeding for 50% of the lambs with seasonally available diets and sell them at heavier carcass weights, offering us 22 million extra kg of lamb carcasses? Or is it worth joining another 800,000 ewes to produce 1 million extra lambs and deliver at 22 kg carcass weights after feeding for 5 to 6 months? The latter strategy involves many additional risks along the value chain. The former strategy may allow producers to obtain better market prices due to out of season sales as we observed in method 2.

It is also important to consider the health and wellbeing of an additional 800,000 ewes to be mated to produce 1 million extra lambs [22, 23], the residual effects of pesticides and worm treatment application for the additional 1 million newly born lambs [24–26] and environmental impacts such as CH_4_ emissions from animals and other emissions associated with the transport for the extra 1 million lambs produced for slaughter [27–29]. The proposed CH_4_ emissions calculation suggest that the extended feeding approach may be more sustainable to the ecosystem potentially delivering 22 million extra kg of lamb carcasses per year to fulfil the growing international demand for red meat from Australia. Furthermore, in the present study for the calculation of 3 weeks of extended feeding, we believe that CH_4_ emissions were expected to be lower. This occurred because the lambs were fed with formulated rations having 45% hay and 55% concentrate (barley, oat and lupin grains) while CH_4_ emissions were calculated from emission rate obtained for sheep under pasture grazing published recently [12]. The inclusion of grain in ruminant diets has been shown to reduce CH_4_ emissions compared with diets containing only pasture or roughage diets high in structural carbohydrates (high fibre) [30]. This means the actual difference in estimated emissions between each of the 4 scenarios and the option of feeding 11 million lambs for an extra 3-week might be even greater than estimated largely due to the latter being reduced, rather than the 4 alternative scenarios having greater emissions (Fig. 3).

It is unlikely all 22 million lambs would be fed for an extended period due to insufficient feed availability and market requirement for meat, but a combination of extended feeding coupled with additional ewes mated is the most likely scenario. Based on the growth rate observed for Composite lambs, if an additional 2 kg carcass weight per animal can be gained for 50% of the current 22 million lambs slaughtered in Australia (= 11 million animals) by 3 weeks of extended feeding, it would possibly supply an additional 22 million kg carcass produced per annum. This is equivalent to producing an extra 1 million lamb carcasses per annum weighing 22 kg each. Alternatively, if we feed 25% of the 22 million lambs (= 5.5 million animals) for 6 weeks of extended feeding resulting in an extra 4 kg carcass weight gain, we would produce the equivalent amount of extra 1 million lamb carcasses per annum weighing 22 kg each. Feeding Composite lambs for an extended period and selling Merino yearlings pre-summer is the likely option due to the faster growth potential of Composite lambs, which may offer greater profit due to quicker turn-over to market. It also offers producers the flexibility to select new stock for replacement of reproduction flock from additional ewes mated yearly. Taking the present experiment as an example, although the growth rate was not linear over the feeding period, feeding Composite lambs for an extra 2–3 weeks would result in an additional 2 kg carcass weight per animal.

The authors note that in this paper, the estimates of economic benefits and CH_4_ emissions from different production systems were based on relatively simple theoretical methods. In future, more comprehensive economic and CH_4_ emission modelling will be completed. This will investigate the cost of producing the raw materials to produce the supplementary diets reported and account for the economic cost and environmental impacts of sowing and harvesting supplementary feedstuffs, depreciation on infrastructure/assets along with the cost of additional labor required to feed the diets to stock. The data presented also does not account for opportunity costs associated with having capital invested in animals and feed. However, the authors believe the cost of these inputs would be close to neutral as producers would be required to source feeds and resources either for producing additional new lambs for the next season or extended feeding of a portion of existing lambs. It is reasonable to state that the extended feeding strategy has more advantage and is less costly, when compared with the production of new additional lambs because it does not require joining of dams, the cost of artificial insemination, nor costs associated with maintenance of pregnant animals, miscarriage and loss of lambs during birth, labour for looking after newly born animals up to weaning and veterinary services and treatments.

## Implications

With the rapid changes in global agriculture and food trade, maintaining a sustainable animal production strategy that protects the ecosystem from harmful or detrimental impacts is essential. Applying an extended feeding strategy to supply meat for national and international demand for high quality animal protein would be a better option than producing another 1 million new lambs by joining an additional 800,000 ewes every year. Nevertheless, we acknowledge a combination of joining additional ewes and extended feeding strategies is the most likely scenario as producers also need to mate ewes for the replacement of reproduction flocks. In the future, the intensification of animal production systems needs to be considered in a broad sense integrating the knowledge and evidence based scientific approach, with the aims of reducing the environmental, social and/or economic impacts to ecosystems and improving animal wellbeing.

## Conclusions

The extended 9-week feeding in the current experiment allowed Composite lambs and Merino yearlings to further grow 17.6–20.3 kg and 10.7–12.9 kg, respectively that resulted in increases in carcass weights of 8.3–9.5 kg and 4.9–5.7 kg, respectively. Composite lambs fed CAMM and CAMH had 5% greater carcass weights at slaughter compared to the CONT group, but dietary treatments did not change carcass weight of Merino yearlings. This in turn offered producers of composite lambs an opportunity to gain extra AUD $20.00 to $25.00 when yearly average prices were used and AUD $40.00 to $47.70 when pre- and post-summer average prices in Victoria were used per Composite lamb. In contrast, Merino yearlings resulted in a loss of AUD -$4.60 to $8.70 when yearly average prices were used and a profit of $2.25 to $5.50 when pre- and post-summer average prices were used. Among the Composites, the profit for animals fed the CAMH and CAMM were AUD $2.75 to $4.50 greater than CONT group when full year average prices were applied while AUD $3.50 to $5.50 greater than CONT group when pre- and post-summer average prices were applied. Although the growth rate observed for composite lambs during the feeding period tested was not linear, based on the carcass weights obtained in this study, feeding Composite lambs for an additional 3-week can offer a minimum of 2 kg extra carcass weight per animal. However, the likelihood of all 22 million lambs being fed for an extended period of 3 weeks is unlikely due to insufficient feed availability and the market requirement for meat but a combination of extended feeding coupled with additional ewes mated is the most likely scenario. The results indicate that the extended feeding approach is more sustainable to the ecosystems potentially delivering 22 million kg of extra lamb carcasses per year to fulfil the growing international demand for red meat production in Australia.

## Supplementary information

**Additional file 1 Supplementary Table 1.**

## Data Availability

The dataset analysed during the current study will be available from the corresponding author on reasonable request, after timely full publication of results from other aspects of the experiment.

## Abbreviations

CAMH: Camelina hay
CAMM: Camelina meal
CONT: Control pellet
DP: Dressing percentage
ME: Metabolisable energy
CP: Crude protein
DM: Dry matter
COMP: Composites
LL: Longissimus lumborum
NIR: Near infrared
MLA: Meat and Livestock Australia
DMI: Dry matter intake
AEC: Animal Ethics Committee
CH_4_: Methane
