# Supplementary material for: An alternative approach for sustainable sheep meat production: implications for food security
Source: J Anim Sci Biotechnol. 2020 Jul 15;11:83. doi: 10.1186/s40104-020-00472-z (PMC7362406; doi:10.1186/s40104-020-00472-z)
Supplement: Supplementary file 1 — Additional file 1 Supplementary Table 1. [file 40104_2020_472_MOESM1_ESM.docx]

**Supplementary Table 1.** Carcass weight classification as trade lamb (18-22 kg), heavy lamb (22-24 kg) and extreme heavy lamb (> 25 kg) from Maternal composite (Composite) lambs and hogget (18-24 kg) from Merino yearlings fed camelina meal (CAMM), camelina hay (CAMH) supplemented pellet diet or control pellet diet (CONT) ^#^

| Items^@^ | Composite lambs | | |  | Merino yearlings (hogget) | | |  |
| --- | --- | --- | --- | --- | --- | --- | --- | --- |
|  | CONT | CAMH | CAMM |  | CONT | CAMH | CAMM |  |
| Total carcass numbers | 32 | 24 | 23 |  | 32 | 24 | 24 |  |
| Carcass weight range, 18 – 22 kg | 5 | 4 | 2 |  | 7 | 3 | 5 |  |
| Carcass weight range, 22 – 25 kg | 20 | 5 | 8 |  | 21 | 17 | 15 |  |
| Carcass weight range, above 25 kg | 7 | 15 | 13 |  | 4 | 4 | 4 |  |
| Carcasses between 18 - 22 kg, % | 15 | 16 | 8 |  | 21 | 12 | 20 |  |
| Carcasses between 22 - 25 kg, % | 63 | 21 | 35 |  | 66 | 71 | 63 |  |
| Carcass greater than 25 kg, % | 22 | 63 | 57 |  | 13 | 17 | 17 |  |

^#^Statistical comparisons were not performed for carcass weight classification as the numbers were counted from each treatment and the percentage was calculated based on total number of lambs and yearlings slaughtered in each treatment. One lamb from camelina meal (CAMM) treatment group was removed a day prior to slaughter due to an injury during blood sample collection
